# Correction: Long-term protection of HPV test in women at risk of cervical cancer

**DOI:** 10.1371/journal.pone.0243000

**Published:** 2020-11-20

**Authors:** The PLOS ONE Staff

In Fig 2, the titles of the y-axes and the A and B labels for the graphs are missing. The publisher apologizes for the error. Please see the correct Fig 2 here.

**Fig 2:**
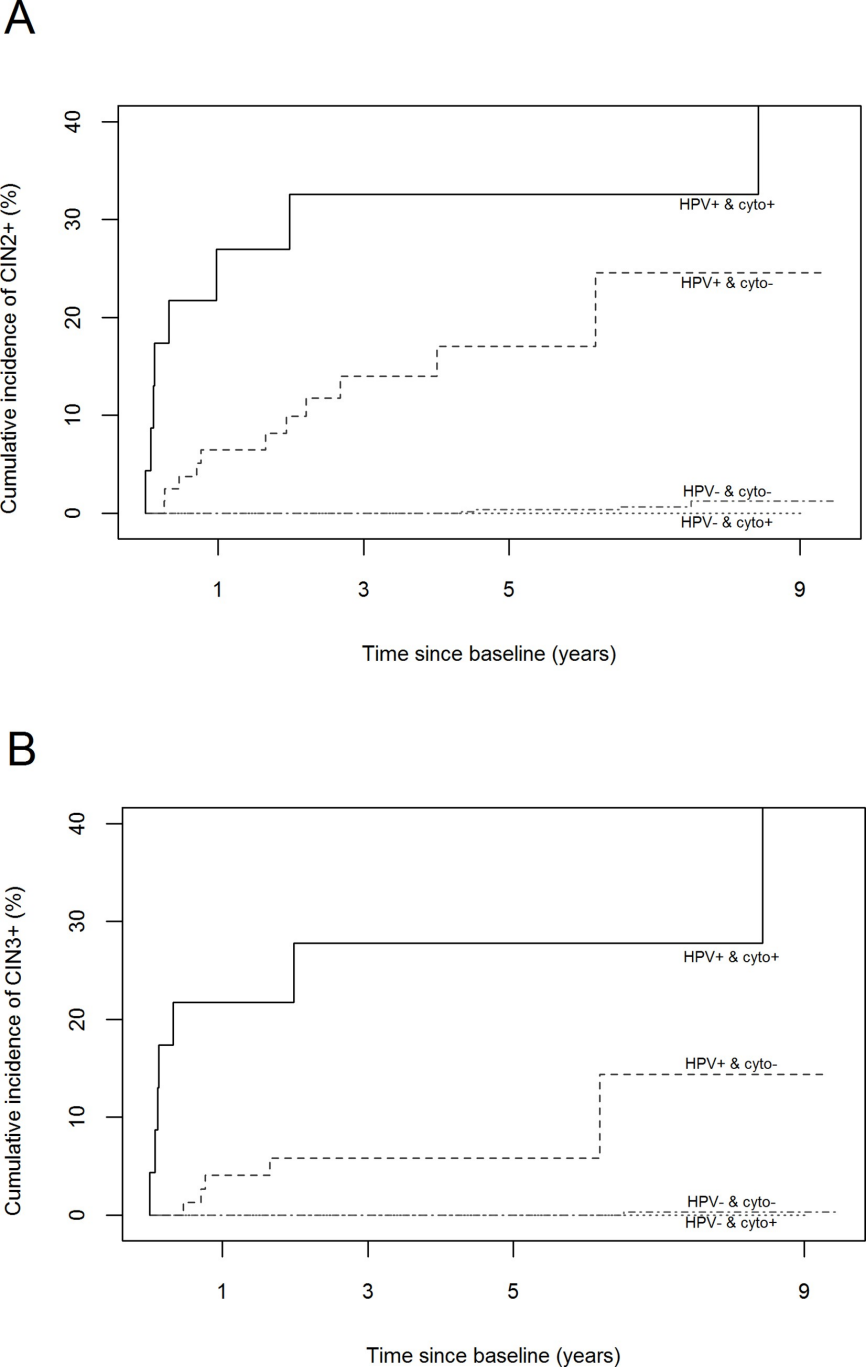
Cumulative incidence of developing a histologically confirmed CIN2+ (A) or CIN3+ (B) by baseline co-testing results among underscreened women. Underscreened women are defined as women older than 39 years with no records on cervical cytology during the previous 5 years. CIN2+ included cervical intraepithelial neoplasia grade 2 and 3 and cervical carcinoma. CIN3+ included cervical intraepithelial neoplasia grade 3 and cervical carcinoma.
