# Supplementary material for: Long-term protection of HPV test in women at risk of cervical cancer
Source: PLoS One. 2020 Aug 27;15(8):e0237988. doi: 10.1371/journal.pone.0237988 (PMC7451648; doi:10.1371/journal.pone.0237988)
Supplement: S1 Table — CIN2+ included cervical intraepithelial neoplasia grade 2 and 3 and cervical carcinoma. CIN3+ included cervical intraepithelial neoplasia grade 3 and cervical carcinoma. The CIN2+ and CIN3+ incidence rate was calculated in person-year per 100 women. (PDF) [file pone.0237988.s001.pdf]

| Screening tests results | Person-years | Number of CIN2+ | Annual incidence rate of CIN2 + per 100 women/year | 95% Conf. Interval | | Number of CIN3+ | Annual incidence rate of CIN3 + per 100 women/year | 95% Conf. Interval | |
|---|---|---|---|---|---|---|---|---|---|
| **HPV positive - cyto positive** | | | | | | | | | |
| **Period of time (years)** | | | | | | | | | |
| (0 - 3] | 40.9 | 7 | 17.1 | 6.9 | 35.2 | 6 | 14.7 | 5.4 | 31.9 |
| (0 - 5] | 52.2 | 7 | 13.4 | 5.4 | 27.6 | 6 | 11.5 | 4.2 | 25 |
| (0 - 9] | 59.3 | 8 | 13.5 | 5.8 | 26.6 | 7 | 11.8 | 4.8 | 24.3 |
| **HPV positive - cyto negative** | | | | | | | | | |
| **Period of time (years)** | | | | | | | | | |
| (0 - 3] | 175.9 | 9 | 5.1 | 2.3 | 9.7 | 4 | 2.3 | 0.6 | 5.8 |
| (0 - 5] | 231.7 | 10 | 4.3 | 2.1 | 7.9 | 4 | 1.7 | 0.5 | 4.4 |
| (0 - 9] | 268.4 | 11 | 4.1 | 2.1 | 7.3 | 5 | 1.9 | 0.6 | 4.4 |
| **HPV negative - cyto positive** | | | | | | | | | |
| **Period of time (years)** | | | | | | | | | |
| (0 - 3] | 27 | 0 | 0 | . | . | 0 | 0 | . | . |
| (0 - 5] | 41.3 | 0 | 0 | . | . | 0 | 0 | . | . |
| (0 - 9] | 56.1 | 0 | 0 | . | . | 0 | 0 | . | . |
| **HPV negative - cyto negative** | | | | | | | | | |
| **Period of time (years)** | | | | | | | | | |
| (0 - 3] | 2467.4 | 0 | 0 | . | . | 0 | 0 | . | . |
| (0 - 5] | 3684.9 | 2 | 0.1 | 0 | 0.2 | 0 | 0 | . | . |
| (0 - 9] | 4693.4 | 4 | 0.1 | 0 | 0.2 | 1 | 0 | 0 | 0.1 |
